# The association between anion gap and in-hospital mortality of post-cardiac arrest patients: a retrospective study

**DOI:** 10.1038/s41598-022-11081-3

**Published:** 2022-05-06

**Authors:** Jun Chen, Chuxing Dai, Yang Yang, Yimin Wang, Rui Zeng, Bo Li, Qiang Liu

**Affiliations:** 1 The Third Affiliated Hospital, Zhejiang Chinese Medical University, Hangzhou, 310000 Zhejiang China; 2 The First School of Clinical Medicine, Zhejiang Chinese Medical University, Hangzhou, 310000 Zhejiang China; 3 Department of Intensive Care Unit, The Fourth Hospital of Hebei Medical University, Shijiazhuang, China

**Keywords:** Biomarkers, Cardiology, Diseases, Medical research, Risk factors

## Abstract

We aimed to determine the association between anion gap and in-hospital mortality in post-cardiac arrest (CA) patients. Extracted the data of patients diagnosed with CA from MIMIC-IV database. Generalized additive model (GAM), Cox regression and Kaplan–Meier survival analysis were used to demonstrate the association between AG levels and in-hospital mortality. ROC curve analysis for assessing the discrimination of AG for predicting in-hospital mortality. Totally, 1724 eligible subjects were included in our study finally. 936 patients (551 males and 385 females) died in hospital, with the prevalence of in-hospital mortality was 54.3%. The result of the Kaplan–Meier analysis showed that the higher value of AG had significant lower survival possibility during the hospitalization compared with the lower-value of AG patients. In the crude Cox regression model, high-level of AG subjects was associated with significant higher HR compared with low-level of AG subjects. After adjusted the vital signs data, laboratory data, and treatment, high-level of AG (group Q3 and group Q4) were also associated with increased risk of in-hospital mortality compared with low-level of AG group, 1.52 (95% Cl 1.17–1.85; P < 0.001), 1.64 (95% Cl 1.21–2.08; P < 0.001), respectively. The ROC curve indicated that AG has acceptable discrimination for predicting in-hospital mortality. The AUC value was found to be 0.671 (95% CI 0.646–0.698). Higher AG levels was associated with poor prognosis in post-CA patients. AG is a predictor for predicting in-hospital mortality of CA, and could help refine risk stratification.

## Introduction

Cardiac arrest (CA) is not uncommon worldwide, approximately 292,000 adults suffer an in-hospital cardiac arrest (IHCA)^1^, and 420,000 people suffer an out-of-hospital cardiac arrest (OHCA) in the United States each year^2^. Recently, one study based on the nationwide emergency department sample of the United States found that the in-hospital survival rate for all CA was only 28.7%, in addition, the incidence of CA is increasing year by year^3^. Lactate, a product of anaerobic glycolysis under tissue ischemia, is generally elevated in patients with sudden cardiac death, and its level increases with the duration of ischemia and hypoxia^9^. Previous studies have reported that the higher level of lactate tends to be associated with a worse prognosis of cardiac arrest patients^5–7^. A decrease in lactate is a surrogate marker for adequate tissue perfusion after the return of spontaneous circulation (ROSC) and potentially serves as an endpoint for resuscitation^4^.

Anion gap (AG) was defined as the difference between unmeasured anions and unmeasured cations, the calculated formula was derived from the concentrations of three commonly measured ions (Na^+^, Cl^−^, HCO^−^_3_)^15^. As a biomarker for assessing the acid–base of the biotic internal environment, but the correlation between AG and lactate was not very clear. One study reported that serum AG was poor in predicting a lower level of lactate (> 2 mmol/L), but has an acceptable capacity for predicting higher lactate levels (> 4 mmol/L)^9^. These results may suggest that there is a nonlinear positive correlation between AG and lactate. In addition, relevant researches on AG in predicting the prognosis of CA was limited. Therefore, we aimed to investigate the association between serum AG levels and serum lactate levels, and determined the association between AG and in-hospital mortality of cardiac arrest patients. Also, we wanted to evaluate the predictive value of AG contributing to in-hospital death of acute cardiac arrest patients and compared the discrimination with lactate.

## Methods

### Data source

This was a retrospective observational study, and the primary data of our study was derived from the MIMIC-IV database (version 1.0). MIMIC-IV database is an extensive database and contained all medical record numbers corresponding to patients admitted to an intensive care unit (ICU) or the emergency department between 2008 and 2019 in the Beth Israel Deaconess Medical Center (BIDMC)^10^. The version 1.0 is the lasted version of the MIMIC-IV database. One of our authors (C.J, certification ID: 8979131) gained permission to document the database after online training at the National Institutes of Health (NIH). Our research was conducted entirely on publicly available, anonymized data, therefore, individual patient consents were not required. All methods were carried out in accordance with relevant guidelines to protect the privacy of patients.

### Population selection

We included adult patients (aged > 18 years old) diagnosed with cardiac arrest at hospital admission by the International Classification of Diseases version 9 and 10 diagnosis codes (“4275”, “I46”, “I462”, “I468”, “I469”) in the MIMIC-IV database. The exclusion criteria were: (I) Missing survival outcome data; (II) During pregnancy and the postpartum period; (III) Incomplete or unobtainable documented or other vital medical data records; (IV) Missing the data of AG.

### Clinical and laboratory data

Patients’ baseline characteristics (age, height, weight, diabetes history, hypertension history, chronic lung disease history, myocardial infarction, heart failure history) were collected. The first document of vital signs data and laboratory tests data of cardiac arrest patients admitted to the hospital were extracted. Vital signs data included body temperature (T), systolic blood pressure (SBP), diastolic blood pressure (DBP), mean blood pressure (MBP), heart rate (HR), respiratory rate (RR), pulse oximetry derived oxygen saturation (spo2). Laboratory tests data included creatinine, AG, PH, lactate, blood urea nitrogen (BUN), chloride, glucose, hemoglobin, hematocrit, white blood cell count, platelet count, serum potassium, serum sodium, and International Nominal Ratio (INR). Therapy included the use of vasoactive drugs (norepinephrine), and continuous renal replacement therapy (CRRT) during the hospitalization were also recorded. The acute physiology score II (APSII)^11^ and sequential organ failure assessment (SOFA) score^12^ were also calculated for each patient. The endpoint of our study was in-hospital mortality which was defined as survival status at the hospital discharge.

### Statistical analysis

The generalized additive model (GAM) was used to demonstrate the correlation between the anion gap and in-hospital mortality in cardiac arrest patients and then the included patients were equally divided into four groups. Continuous variables that exhibited a normal distribution were documented as the mean ± standard deviation (SD). Otherwise, they were documented as medians with upper and lower quartiles. Categorical variables were documented as frequencies with percentages. Group comparisons were pooled using Chi-square, 1-way ANOVA, and Kruskal–Wallis tests. Pearson correlation analysis was used to analyze the correlation among anion gap, lactate, and PH. Kaplan–Meier survival analysis was used to pool the difference of in-hospital mortality between the various groups and analyzed by Log-rank test. Variables based on epidemiological and laboratory test indicators may exist as potential confounders^14^. For clinical outcome, three Cox proportional risk regression models were constructed based on the basis of anion gap group inclusion according to quartile. The first quartile was treated as the reference group. In the model I, covariates were mainly adjusted for vital signs data and comorbidities (age, MBP, spo2, hypertension, diabetes, chronic pulmonary disease, heart rate, respiratory rate, temperature). In the model II, covariates were mainly adjusted for laboratory data (albumin, creatinine, PH, lactate, glucose, hemoglobin, platelet count, INR, white blood cell count) based on the model I. In the model III, covariates were mainly adjusted for treatment (CRRT) and disease severity score (SOFA score, apsiii score) based on the model II. The discrimination of anion gap for in-hospital mortality was assessed by receiver operating characteristic (ROC) curve analysis. The area under the curve (AUC) of the ROC curve more than 0.7 was regarded as good discrimination, 0.65–0.70 represented moderate discrimination. These results were expressed as hazard ratio with 95% confidence intervals (CIs). All tests were 2-tailed tests, and p ≤ 0.05 was considered statistically significant. Statistical analyses were performed using R version 3.6.3 (R Foundation for Statistical Computing, Vienna, Austria).

### Ethics approval and consent to participate

Our research was conducted entirely on publicly available, anonymized data, therefore, individual patient consents were not required. All methods were carried out in accordance with relevant guidelines to protect the privacy of patients.

## Results

### The characteristics of study patients

Totally, 1724 eligible patients (1055 males and 669 females) with an average age of 66.34 ± 16.16 years old were included in our study finally, More details about the data extraction process and missing data as shown in Supplementary Tables S1 and S2. 936 patients (551 males and 385 females) died during the hospitalization, with the incidence of in-hospital mortality was 54.3%. Patients were evenly divided into four groups according to the GAM for anion gap and in-hospital mortality. The baseline characteristics of these patients were summarized in Table 1. Patients with a higher level of anion gap presented a higher level of BUN, creatinine, blood glucose, potassium, and INR. In terms of vital signs, patients with higher level of anion gap tend to have lower blood pressure, SPO_2_, and temperature, however, they heart rates and respiratory rate increased significantly. In terms of severity of disease scoring, a higher level of anion gap patients received higher scores, it is interesting to note there was no significant difference in APSII scores among the groups. While, the use of norepinephrine and CRRT were also more common in the higher-level of anion gap group than the lower-level of anion gap group. Also, patients with a higher anion gap have longer ICU and hospital stays than patients with the lower anion gap group.

**Table 1:** The characteristic of included subjects.

Characteristic	Q1 (n = 431)	Q2 (n = 431)	Q3 (n = 431)	Q4 (n = 431)	P value
Age (years old)	67.13 ± 15.90	66.56 ± 16.16	66.31 ± 16.55	65.43 ± 16.50	0.486
Man	269 (62.41%)	254 (58.93%)	258 (59.86%)	274 (63.57%)	0.467
SBP (mmHg)	113.94 ± 14.42	115.23 ± 16.03	112.03 ± 16.91	108.25 ± 19.76	< 0.001
DBP (mmHg)	61.16 ± 10.40	63.71 ± 11.43	61.53 ± 12.01	60.71 ± 13.25	< 0.001
MBP (mmHg)	76.96 ± 10.08	79.02 ± 11.20	76.08 ± 11.99	73.71 ± 13.37	< 0.001
Heart rate (beats/minute)	83.57 ± 17.89	83.95 ± 18.56	87.33 ± 18.71	90.13 ± 18.73	< 0.001
Respiratory rate (beats/minute)	19.91 ± 4.00	20.55 ± 4.21	21.48 ± 4.47	22.66 ± 4.95	< 0.001
Temperature (°C)	36.69 ± 0.80	36.59 ± 0.99	36.46 ± 1.10	36.16 ± 1.26	< 0.001
SPO_2_ (%)	97.29 ± 3.88	97.21 ± 3.28	96.07 ± 6.47	94.06 ± 7.96	< 0.001
**Comorbidities, n (%)**					
Diabetes	123 (28.54%)	160 (37.12%)	149 (34.57%)	186 (43.16%)	< 0.001
Hypertension	185 (42.92%)	178 (41.30%)	169 (39.21%)	110 (25.52%)	< 0.001
Myocardial infarction	111 (25.75%)	128 (29.70%)	132 (30.63%)	132 (30.63%)	0.338
Congestive heart failure	165 (38.28%)	184 (42.69%)	194 (45.01%)	190 (44.08%)	0.196
Chronic pulmonary disease	128 (29.70%)	138 (32.02%)	111 (25.75%)	86 (19.95%)	< 0.001
**Laboratory parameters**					
Albumin (g/dL)	3.00 (2.48–3.50)	3.10 (2.70–3.70)	3.15 (2.60–3.60)	3.00 (2.45–3.00)	0.473
Anion gap (mEq/L)	12.00 (6.50–13.5)	15.00 (14.00–16.50)	18.00 (17.00–20.00)	23.50 (20.50–43.00)	< 0.001
BUN (mg/dL)	23.70 ± 14.67	29.00 ± 19.30	35.46 ± 22.35	47.14 ± 32.35	< 0.001
Bicarbonate (mmol/L)	24.36 ± 4.73	22.02 ± 3.96	20.29 ± 3.99	16.54 ± 4.58	< 0.001
Creatinine (mg/dL)	1.11 ± 0.62	1.49 ± 1.00	2.01 ± 1.51	3.21 ± 3.08	< 0.001
Chloride (mmol/L)	105.96 ± 5.86	105.17 ± 6.12	103.42 ± 6.53	100.78 ± 6.90	< 0.001
Glucose (mg/dL)	156.71 ± 74.87	177.03 ± 69.10	196.68 ± 83.08	225.11 ± 126.99	< 0.001
Hematocrit (%)	31.60 ± 6.11	34.28 ± 7.00	33.47 ± 7.29	32.53 ± 7.56	< 0.001
Hemoglobin (g/dL)	10.32 ± 2.14	11.18 ± 2.44	10.87 ± 2.48	10.39 ± 2.51	< 0.001
INR	1.50 ± 0.87	1.58 ± 0.92	1.78 ± 1.17	2.16 ± 1.51	< 0.001
Lactate (mmol/L)	2.46 ± 1.61	3.06 ± 1.88	3.91 ± 2.05	7.12 ± 4.06	< 0.001
Platelet (10^9^/L)	207.33 ± 111.48	212.25 ± 95.36	216.52 ± 103.18	193.44 ± 112.46	0.009
PH	7.35 ± 0.09	7.33 ± 0.09	7.30 ± 0.11	7.22 ± 0.14	< 0.001
Potassium (mmol/L)	4.30 ± 0.67	4.26 ± 0.63	4.43 ± 0.72	4.67 ± 0.88	< 0.001
Sodium (mmol/L)	139.06 ± 4.50	139.21 ± 5.00	138.35 ± 5.53	138.58 ± 6.49	0.042
WBC (10^9^/L)	13.25 ± 6.99	13.93 ± 8.66	14.93 ± 7.17	10.56 ± 9.84	< 0.001
Renal replacement therapy, n (%)	23 (5.34%)	30 (6.96%)	50 (11.60%)	99 (22.97%)	< 0.001
Norepinephrine, n (%)	216	243	303	349	< 0.001
**Scoring systems**					
SOFA	7.68 ± 3.88	8.42 ± 4.03	10.03 ± 3.85	11.87 ± 4.04	< 0.001
APSII	71.70 ± 31.23	75.48 ± 29.30	73.55 ± 30.93	70.31 ± 27.67	0.062
ICU LOS, days	6.82 ± 8.01	6.72 ± 7.54	6.45 ± 8.74	4.81 ± 6.88	< 0.001
HOS LOS (days)	20.45 ± 19.37	16.77 ± 17.58	14.28 ± 15.72	10.67 ± 15.07	< 0.001
HOS mortality, n (%)	166 (38.52%)	189 (43.85%)	260 (60.32%)	321 (74.48%)	< 0.001

*SBP* systolic blood pressure, *DBP* diastolic blood pressure, *MBP* mean blood pressure, *SPO*_*2*_ pulse oximetry derived oxygen saturation, *BUN* blood urea nitrogen, *INR* international nominal ratio, *WBC* white blood cell, *SOFA* sequential organ failure assessment, *APSII* acute physiology score II, *ICU* intensive care unit, *HOS* hospital, *LOS* length of stay.

### Anion gap levels and in-hospital mortality of CA

**Figure 1:**
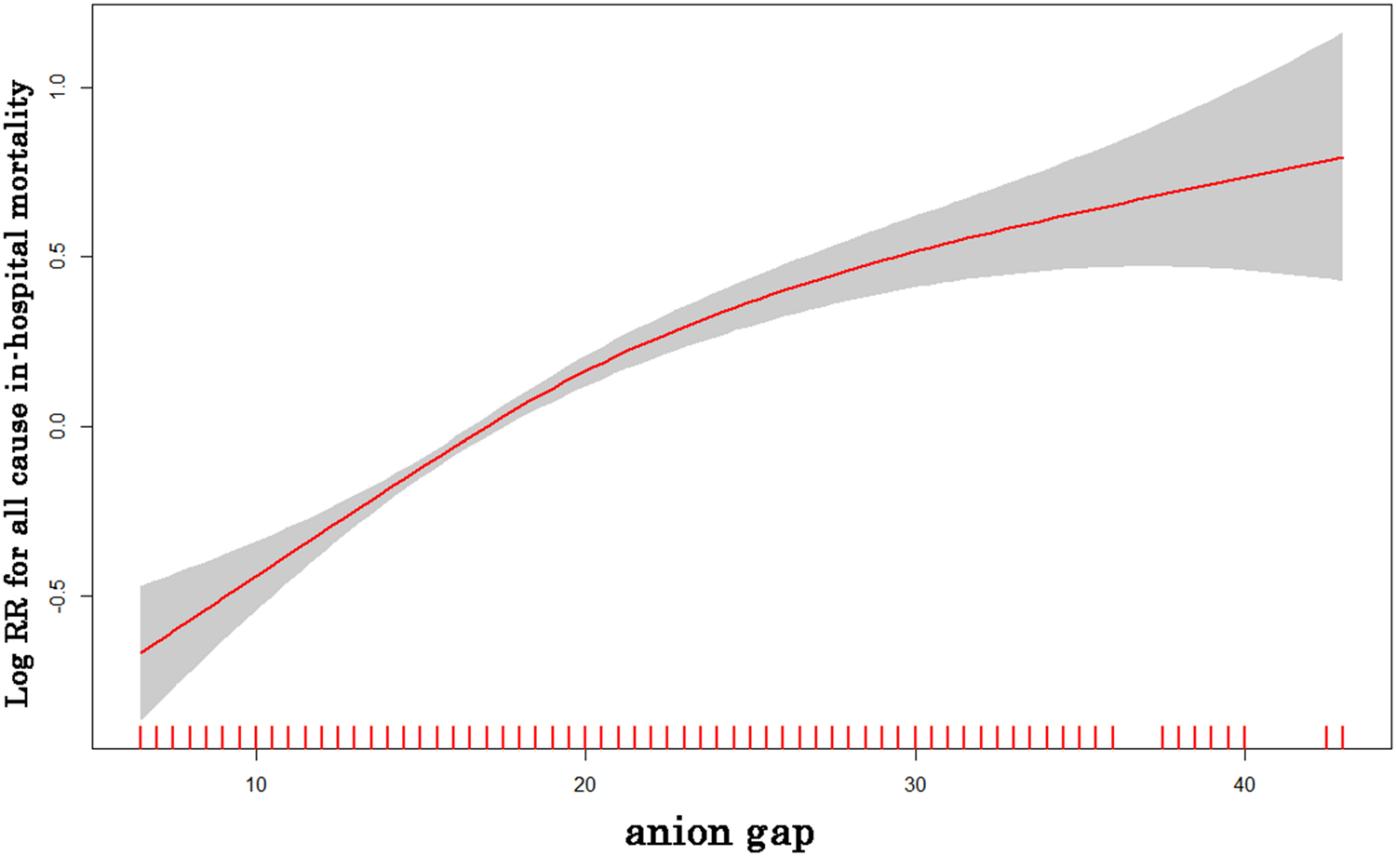
Cubic spline plot of relation of anion gap to risk of inpatient mortality. The model is fitted using restricted cubic splines with four knots in the generalized additive model. The ordinate represents log (RR) of in-hospital mortality. The abscissa represents the level of anion gap. The solid line represents the relationship between log (RR) of in-hospital mortality and admission anion gap level, and shaded area represents the 95% CI.

**Figure 2:**
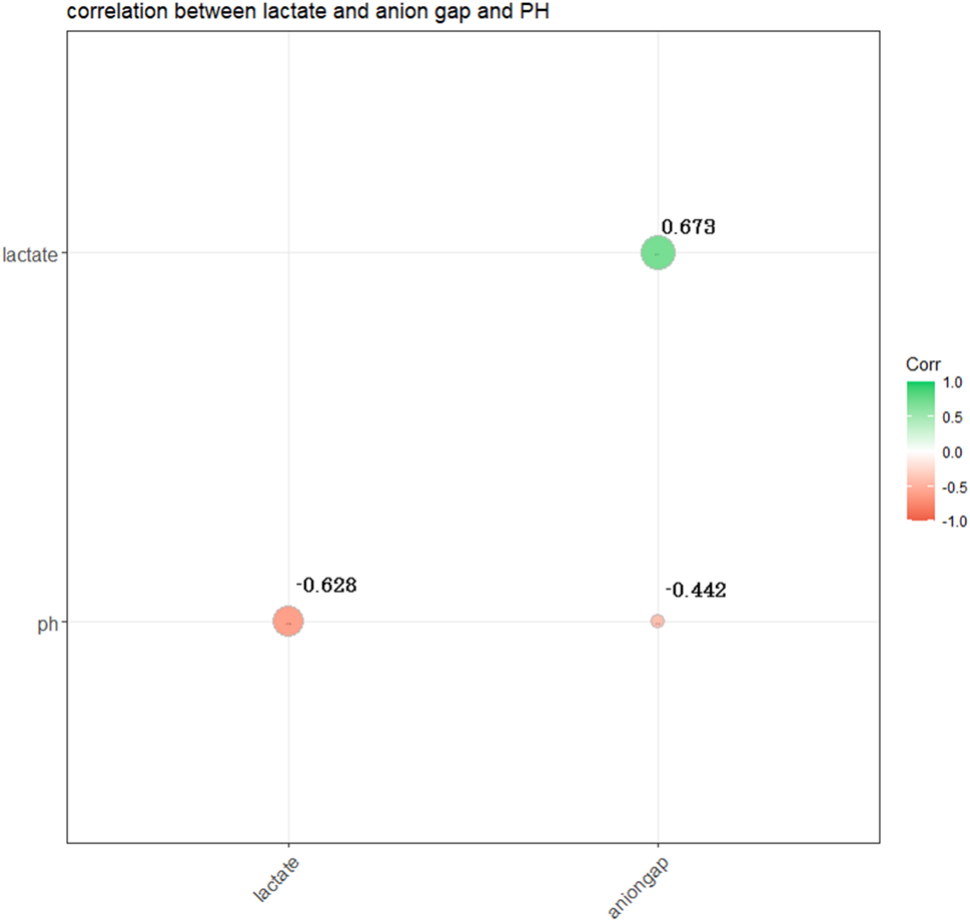
The correlation between serum AG level and serum lactate level and pH value.

The generalized additive model (GAM) showed that a positive but nonlinear correlation between AG and in-hospital mortality (Fig. 1). In the unadjusted Cox regression model, high-level AG was associated with higher in-hospital mortality compared with low-level AG (HR of Q2 vs Q1: 1.30; HR of Q3 vs Q1: 1.98; HR of Q4 vs Q1: 3.16). We used three Cox regression models to adjust other confounding factors and further determine the association between AG and in-hospital mortality of CA patients (Table 2). In the model I, after adjusting vital signs data and comorbidities, a high-level of AG was associated with an increased risk of in-hospital mortality (HR of Q2 vs Q1: 1.27; HR of Q3 vs Q1: 1.73; HR of Q4 vs Q1: 2.43). In the model II, covariates were fulled adjusted for laboratory data based on model I, the group of Q3 and Q4 also showed a significantly high risk of in-hospital mortality, and the HR was 1.16, 1.47, and 1.67, respectively. In the model III, covariates were further adjusted for treatment (norepinephrine, CRRT) and severity of disease scoring (APSII scores and SOFA scores) based on model II. The in-hospital mortality risk has remained significantly higher in the high-level AG group. The survival analysis curve for patients with different levels of AG was shown in a Kaplan–Meier analysis plot in Fig. 2. The result showed that the patients with high-levels of AG had lower survival possibilities during the hospitalization than the low-levels of AG subjects, which reached statistical differences (log-rank test: P < 0.001).

**Table 2:** Anion gap levels and all-cause in-hospital mortality of post-cardio arrest patients.

Variable	Crude model		Model I		Model II		Model III	
	HR (95% CI)	P value	HR (95% CI)	P value	HR (95% CI)	P value	HR (95% CI)	P value
Anion gap (Q1)	1.0 (ref)		1.0 (ref)		1.0 (ref)		1.0 (ref)	
Anion gap (Q2)	1.30 (1.05–1.60)	0.015	1.27 (1.03–1.57)	0.025	1.16 (0.87–1.48)	0.239	1.14 (0.75–1.54)	0.477
Anion gap (Q3)	1.98 (1.63–2.41)	< 0.001	1.73 (1.42–2.11)	< 0.001	1.47 (1.18–1.77)	< 0.001	1.52 (1.17–1.85)	< 0.001
Anion gap (Q4)	3.16 (2.62–3.82)	< 0.001	2.43 (1.99–2.96)	< 0.001	1.67 (1.30–2.03)	< 0.001	1.64 (1.21–2.08)	< 0.001

*HR* hazard ratio, *CI* confidence interval. Models were derived from Cox regression models. Crude model adjusted for: none.

Model I adjusted for: age, mean blood pressure, SPO_2_, hypertension, diabetes, chronic pulmonary disease, heart rate, respiratory rate, temperature.

Model II adjusted for: Model I add albumin, creatinine, PH, lacate, hemoglobin, Glucose, Platelet, INR, WBC.

Model III adjusted for: Model I add Model II add CRRT, norepinephrine, SOFA score, apsiii score.

### The relationship between anion gap, lactate and pH value

**Figure 3:**
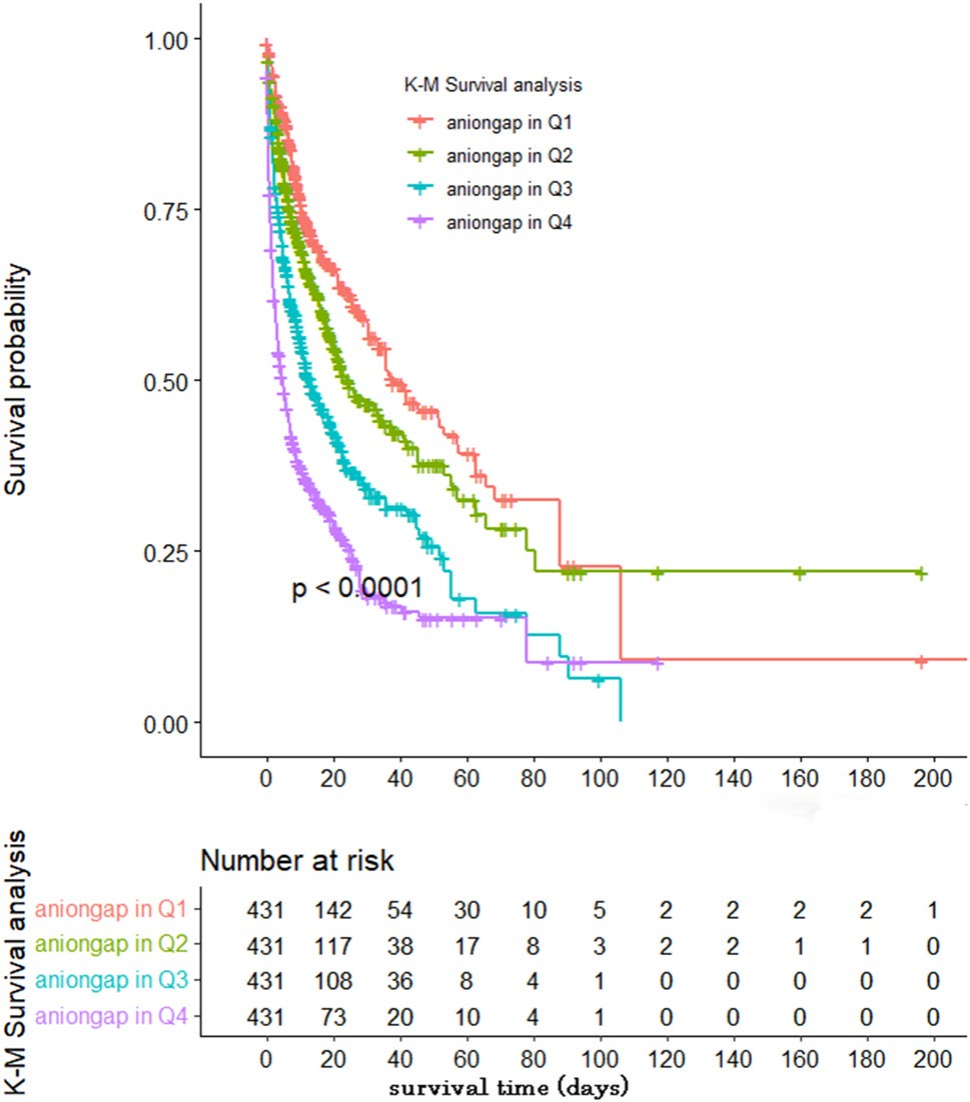
The result of Kaplan–Meier survival curve showed that the high-level of anion gap group had lower survival possibility during the hospitalization than the low-level of anion gap group, which reached statistical differences (log-rank test: P < 0.001).

The pearson correlation analysis showed that there was a strong correlation between lactate and AG (R = 0.673), the correlation between lactate and PH was slightly weaker (R = − 0.628). The correlation between AG and PH was moderate strength (R = − 0.442). (Fig. 3).

### The discrimination of anion gap for predicting in-hospital mortality of CA patients

**Figure 4:**
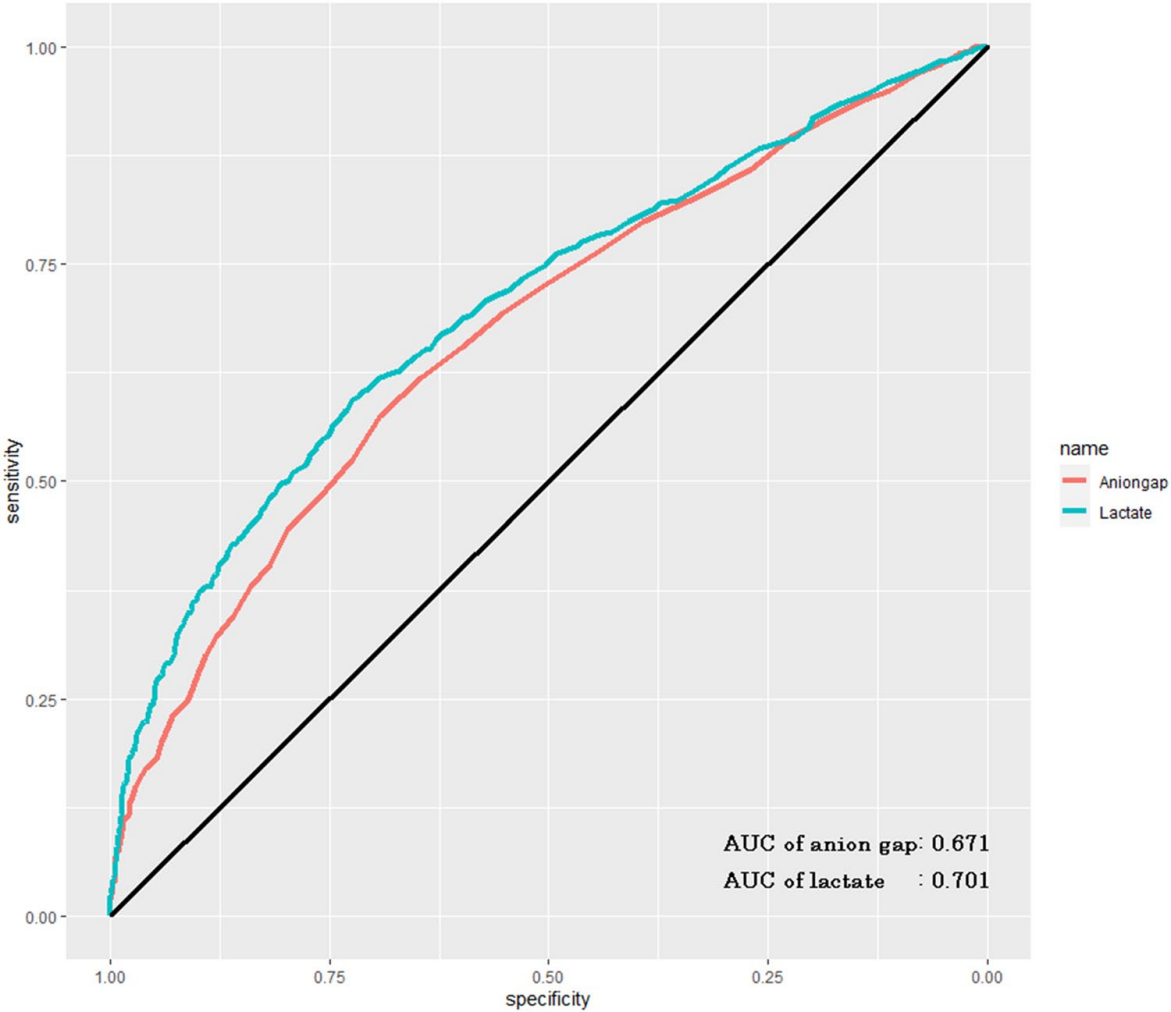
Receiver operating characteristic (ROC) curve of anion gap. The area under the curve (AUC) of ROC of anion gap was 0.671 (95% CI 0.646–0.698). The area under the curve (AUC) was 0.701 (95% CI 0.667–0.735) for ePVS derived from Hakim formula.

The area under the curve (AUC) of the ROC curve was 0.671 (95% CI 0.646–0.698) of AG for predicting in-hospital mortality of CA patients. While, the AUC of lactate was 0.701 (95% CI 0.667–0.735), it indicated that AG has moderate discrimination for predicting in-hospital mortality of CA patients, and lactate has better discrimination for predicting in-hospital mortality of CA patients compared with AG (Fig. 4).

## Discussion

Our study demonstrated a positive but nonlinear correlation between the admission anion gap and in-hospital mortality among CA patients. The total in-hospital mortality was 54.3% in our study, which was similar to the data about 57.8% from the US national inpatient sample^8^. Our main findings were: (1) higher-level AG was independently associated with a higher risk of in-hospital death of CA patients; (2) cardiac arrest patients with higher-level AG have a significantly lower survival possibility during hospitalization than patients with lower-level AG; (3) AG have moderate discrimination for predicting in-hospital death of CA patients; (4) lactate was slightly better than AG in predicting in-hospital death in cardiac arrest patients.

Serum AG is a vague concept based on the difference in serum cation and anion concentrations, which was calculated as [Na^**+**^] − ([Cl^**−**^] + [HCO^−^_3_])^15^. Usually, the unmeasured anions exceed the unmeasured cations, and the AG values range between 8 and 16 mEq/L^21^. In the MIMIC-IV database, the normal AG values range between 8 and 20 mEq/L. In the critical care setting, the AG is of great value in determining metabolic acidosis and is always used for the differential diagnosis of acid–base disorders. Previous studies have reported that high AG was positively correlated with the severity or poor prognosis of many diseases, and could be used to predict the mortality of acute kidney injury, acute myocardial infarction, aortic aneurysm, or even in the elderly general population^16–20^. However, there was limited research on the association between AG and the prognosis of patients who experienced cardiac arrest. To our knowledge, this is the first study to demonstrate that an elevated AG was significantly associated with a higher risk of in-hospital mortality in patients experienced CA.

The status of individuals with cardiac arrest, blood flow was interrupted throughout the body with systemic hypoperfusion, which lead to the metabolism of tissue from aerobic metabolism to anaerobic glycolysis. The lactate is a product or indicator of tissue ischemia and hypoxia^22^. With the prolonged period of ischemia and hypoxia, the level of serum acidic substance was further increased, resulting in the increase of serum AG level. Therefore, the level of serum lactate and serum AG can preliminarily estimated the time of tissue ischemia in patients experienced CA. The time of tissue ischemia is closely related to the poor prognosis including death and neurological complications^23–26^. However, whether serum lactate is superior to serum AG in predicting the in-hospital death of post-CA patients, there were limited reports. In our study, we found that lactate value was a stronger predictor of in-hospital mortality of post-cardiac arrest patients than AG (AUC of ROC 0.701 vs 0.671). We suspected that there were several possible mechanisms. Lactate was the early product of tissue ischemia. With the increase of lactate, the anionic gap value gradually increases. However, compared with lactate, the AG level is affected by more factors. Some studies revealed that renal function can regulate the acid–base balance by regulating the concentration of bicarbonate, which has an important influence on the level of AG^29^. Thus, lactate is more helpful to directly evaluate the severity of post-CA patients compared with the AG. In addition, previous research reported that serum anion gap predicts a lower level of lactate (> 2 mmol/L) poorly^9^. One study proposed that correcting AG for variations in the albumin concentration will improve its sensitivity for predicting a lower level of lactate^28^. We also analyzed the correlation between serum AG level and serum lactate level and pH value. The result found that there was a strong positive correlation between serum AG level and serum lactate level (R = 0.673).

Previous studies reported that serum AG level was associated with renal function^27^. Acute kidney injury (AKI) was common in patients experienced CA. One study reported that about 69.8% of cardiac arrest survivors developed AKI and 69.4% of AKI died in the hospital^13^. In addition, multiple studies have confirmed that renal function is found to be an independent risk factor for in-hospital mortality in critically diseases^30–32^. Considering this point, we used two models to fully adjust the association between AG and in-hospital mortality (model II has adjusted for creatinine, model III has adjusted for CRRT). In the present study, we found that the serum anion gap measured during admission 24 h was significantly associated with poor clinical outcomes after adjusting for the confounding effects of multiple factors. These results suggested that AG was independently associated with a higher risk of in-hospital death of patients who experienced CA.

Few studies have evaluated the utility of blood AG for predicting the in-hospital mortality of post-cardiac arrest patients. Our findings were based on a large sample that included patients with more diverse clinical data. The results showed that serum AG level can be used as a prognostic factor for patients who experienced CA. Although the normal range of anion gaps is currently wide (8 to 16 mEq/L or 8 to 20 mEq/L), monitoring of serum AG in the early stage of post-cardiac arrest can effectively guide fluid resuscitation and correct the acid–base balance of the internal environment. Based on our findings, we found that serum anion gap level over 23 mEq/L (Q4 group) patients have an significantly increased risk of in-hospital death compared with the patients under 13.5 mEq/L (Q1 group). Therefore, for the post-CA patient with serum anion gap level over 23 mEq/L, we may need to positively identify the cause of high serum anionic gap and provide effective treatment. Future studies should explore the value of AG measurement in discrimination of higher risk subgroup of patients experienced CA, and provide more valuable guidance on early goal-directed therapy for fluid resuscitation and acid–base balance of internal environment. Furthermore, AG values can be obtained from blood biochemistry (derive from venous blood), and can be obtained more easily than lactate values (derive from arterial blood gas). Thus, AG can be used as a supplementary biomarker of lactate to guide subsequent therapy.

### Study limitation

Some limitations should be taken into account in our study. First, the present study did not further analyze the causes of cardiac arrest, such as cardiac arrest caused by malignant arrhythmia or myocardial infarction or severe electrolyte disturbance, or other conditions. Future studies can conduct a subgroup analysis of specific causes of cardiac arrest to provide more evidence for this term. Second, our study only researched the in-hospital mortality of the patients with post-cardiac arrest, and future researches can investigate the association between admission AG and long-term prognosis (long-term mortality or cardiovascular complications) in cardiac arrest patients.

## Conclusion

Higher AG levels were associated with poor prognosis in post-cardiac arrest patients. The anion gap is a predictor for predicting in-hospital mortality of post-cardiac arrest patients and could help refine risk stratification.

## Supplementary Information

Supplementary Tables.

## Data Availability

The datasets generated and analyzed during the current study are available from the corresponding author on reasonable request.

## Abbreviations

CA: Cardiac arrest
IHCA: In-hospital cardiac arrest
OHCA: Out-of-hospital cardiac arrest
GAM: Generalized additive model
ICD: International Classification of Diseases
AUC: Area under the curve
ROC: Receiver operating characteristic
Cl: Confidence intervals
SBP: Systolic blood pressure
DBP: Diastolic blood pressure
MBP: Mean blood pressure
SPO2: Pulse oximetry derived oxygen saturation
BUN: Blood urea nitrogen
INR: International nominal ratio
WBC: White blood cell
CRRT: Continuous renal replacement therapy
SOFA: Sequential organ failure assessment
APSII: Acute physiology score II
ICU: Intensive care unit
HOS: Hospital
LOS: Length of stay
