# Supplementary material for: The association between anion gap and in-hospital mortality of post-cardiac arrest patients: a retrospective study
Source: Sci Rep. 2022 May 6;12:7405. doi: 10.1038/s41598-022-11081-3 (PMC9076652; doi:10.1038/s41598-022-11081-3)
Supplement: Supplementary file 1 — Supplementary Tables. [file 41598_2022_11081_MOESM1_ESM.docx]

**Table S1 Number of observations after each selection procedure**

| Step | Criteria | Number excluded | Number of remaining observations |
| --- | --- | --- | --- |
| 1 | Total subjects admissions included in MIMIC-IV database | - | 255106 |
| 2 | Total subjects ICU admissions included in MIMIC-IV database | - | 53150 |
| 3 | Include patients on admission diagnosed with CA (ICD-9 code and ICD-10 code between) and stay in ICU |  | 2186 |
| 4 | Exclude if patients with missing aniongap data information | 36 | 2150 |
| 5 | Exclude if patients with missing lactate data information | 426 | 1724 |
| 6 | Exclude if patients with missing ph data information | 0 | 1724 |

**Table S2 Missing number (%) for characteristics**

| Characteristics | Missing number (%) |
| --- | --- |
| Demographics |  |
| Age | 0 |
| Gender | 0 |
| Ethnicity | 0 |
| Marital status | 0 |
| height | 764 (44.3%) |
| weight | 216 (12.5%) |
| Vital signs |  |
| HR | 6 |
| SBP | 6 (0.3%) |
| DBP | 6 (0.3%) |
| MBP | 6 (0.3%) |
| RR | 6 (0.3%) |
| T | 278 (0.3%) |
| SpO2 | 60 (0.3%) |
| Comorbidities |  |
| diabetes | 0 |
| m1alignant_cancer | 0 |
| myocardial infarction | 0 |
| hypertension | 0 |
| renal_disease | 0 |
| chronic_pul1onary_disease | 0 |
| congestive_heart_failure | 0 |
| Laboratory parameters | 0 |
| aniongap | 0 |
| albumin | 864 (50.1%) |
| bicarbonate | 0 |
| bun | 0 |
| creatinine | 0 |
| chloride | 0 |
| glucose | 4 (0.2%) |
| hematocrit | 3 (0.2%) |
| hemoglobin | 6 (0.4%) |
| lactate | 0 |
| potassium | 0 |
| inr | 37 (2.1%) |
| sodium | 22 |
| wbc | 7 (0.4%) |
| platelet | 7 (0.4%) |
| Scoring system |  |
| SOFA | 0 |
| apsiii | 0 |
| Renal replacement treatment | 0 |
| ICU LOS | 0 |
| In-hospital LOS | 0 |
| In-hospital mortality | 0 |
